# Patellectomy for osteoarthritis: a new tension preserving surgical technique to reconstruct the extensor mechanism with retrospective review of long-term follow-up

**DOI:** 10.1186/s13018-015-0237-1

**Published:** 2015-07-10

**Authors:** Vipin Asopa, Charles Willis-Owen, Greg Keene

**Affiliations:** SPORTSMED.SA, 32 Payneham Road, Stepney, Adelaide, SA 5069 Australia

**Keywords:** Patella, Osteoarthritis, Patellectomy, Anterior, Knee, Pain, Extensor

## Abstract

**Background:**

The management of severe patellofemoral arthritis in young patients remains a significant problem. For many, patellofemoral replacement is not a desirable option. Current surgical techniques for patellectomy disrupt the extensor lever arm causing weakness. We describe a new technique that maintains the extensor mechanism tension and a case series showing good results for patella-only arthritis at a mean follow-up of 11 years.

**Methods:**

Eight patellectomies were performed using a new surgical technique in patients with a mean age of 38 years, and an average follow-up of 11 years (range 8–16 years). Patients were followed up using a pain visual analogue scale, Lysholm knee score and patient-reported outcome measures.

**Results:**

All patients experienced pain relief following surgery. Those with patella-only arthritis had better outcomes than patients who had patella and trochlea disease. All patients had either full or near full extension. Lysholm scores were better in patients who had disease confined to the patella.

**Conclusion:**

We believe patellectomy with this tension-preserving technique has a role for the management of anterior knee pain secondary to severe patella-only arthritis in young patients where arthroplasty is not desirable.

## Background

The management of severe patellofemoral arthritis has remained difficult and controversial. Patellofemoral arthroplasty has poor long-term survival and is declining dramatically in popularity [1]. In 2013, there were only 1109 patellofemoral replacements carried out in the UK representing 1 % of knee arthroplasty procedures [2]. In young patients with disabling patellofemoral osteoarthritis, other treatment options are required.

The patella has an important role in increasing the torque developed by contraction of the quadriceps muscle. It achieves this by acting as a variable lever arm; in extension, the patella rises up and out of the femoral notch, increasing the quadriceps lever arm; following patellectomy, a 30 % increase in torque is needed to hold the leg in full extension [3–7].

The use of patellectomy as a treatment has varied over the past 100 years due to poor reported results. In 1909, Heineck reported poor outcomes in five cases of patellectomy that had developed significant impairment of power and Grelsamer recently referred to patellectomy as being popular only at a time when structures around the knee were thought to be expendable [8, 9]. Patellofemoral arthritis is a difficult problem to treat in young patients. A number of treatment modalities have been attempted in the past with variable success. Lateral release to reduce patellofemoral contact pressures was once popular but has subsequently been shown to have poor results. Patellofemoral arthroplasty remains an appealing option in selected patients; however, these devices have amongst the highest revision rates on both the UK and Australian national joint registries [10, 11]. This makes its use limited in young active patients.

We believe that our newly developed technique for patellectomy is useful for severe patella-only arthritis, in carefully selected patients. The standard techniques for patellectomy involve a longitudinal approach and excision of patella, resulting in an excessive length of the residual extensor mechanism and force going through the retinacula. This can cause stretching and lateral subluxation of the quadriceps patella tendon junction and may be responsible for ongoing pain. Due to lack of tension in the remaining extensor mechanism, power is greatly reduced and extensor lag is common. Other issues include cosmetic dissatisfaction, anterior contact sensitivity, and persistent pain from remaining femoral trochlear arthritis.

A cadaveric study showed that transverse repair with shortening of the extensor mechanism increases the lever arm [3, 12], and recently, another study reported enucleation of the patella with transverse repair to have good results [13]. We believe that restoring the correct extensor mechanism length and tension is the key to a good outcome. Furthermore, it so happens that the correct degree of shortening typically required is equal to the longitudinal length of the patella.

We describe a new technique for patellectomy; the patella, with its surrounding soft tissue, is sharply excised as opposed to enucleated from the tendon, and the remaining patella tendon is repaired directly to the quadriceps tendon. This results in a shortening of the extensor mechanism equal to the patellar length. This has yielded good outcomes for patients with disease confined to the patella in our study.

## Methods

Board ethical approval (Sportsmed.SA) and consent from study participants was obtained. Since 1996 and 2007, six patients (eight knees) underwent patellectomy in our institution by a single surgeon (GK). Our indications for patellectomy were severe ongoing or recurrent anterior knee pain unresponsive to previous arthroscopic debridement, extensive specialised physiotherapy and conservative treatment including restricted bent knee activity and attempted weight loss. Arthroscopy had confirmed advanced osteoarthritis of only the patella or patellofemoral joint (patella and trochlea). Involvement of other compartments excluded patients from patellectomy. In addition, all patients had a bone scan with SPECT to demonstrate severe activity isolated to the patellofemoral joint.

Patellectomy was deemed appropriate for these patients due to their young age (mean age 41). They were considered too young for patellofemoral arthroplasty, which is a procedure we consider more suited for older patients with this condition. All patients were extensively counselled regarding the traditional views of the positives and negatives of patellectomy.

**Fig. 1:**
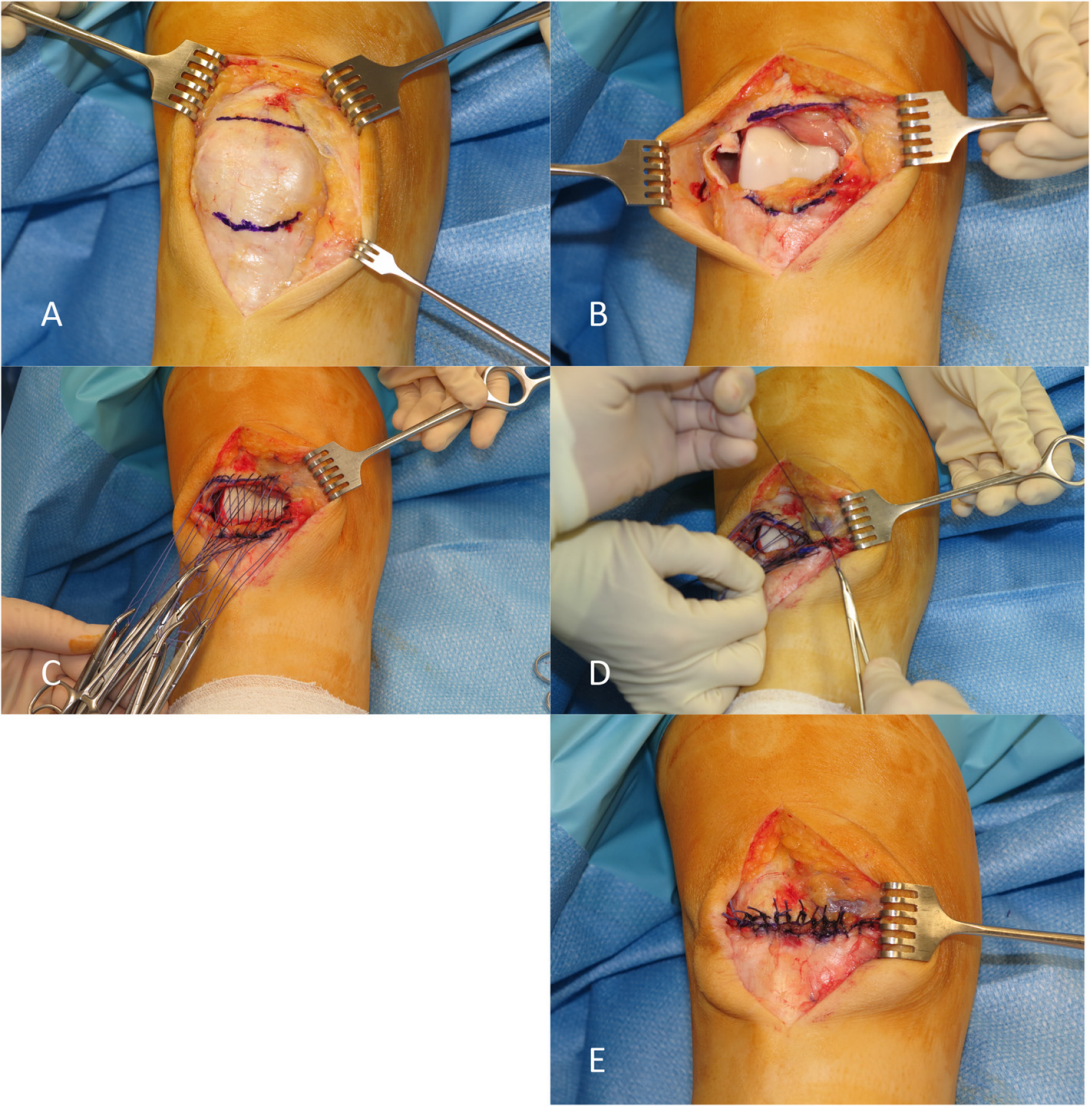
Tension preserving surgical technique for patellectomy. **a** Marking resection of patella. **b** Resection of patella from the quadriceps and patella tendon. **c** Passing suture through the two ends of quadriceps and patella tendon. **d** Progressively tying sutures. **e** Completed extensor mechanism repair

Patellectomy was performed through a vertical midline skin incision. The quadriceps tendon was then resected transversely by sharp dissection directly off its attachment to the superior pole of the patella, and the patella tendon was then similarly transversely resected from the inferior pole of the patella (see Fig. 1). No attempt was made to save the extensor mechanism tissue on the anterior surface of the patella, which is a feature of traditional patellectomy techniques. The two ends of the extensor mechanism were sutured together using a direct end-to-end interrupted suture technique using a strong non-absorbable braided suture (#1 Ti-Cron, Covidien, Ireland). Up to 12 sutures were inserted and held with artery clips but not tensioned or tied until all were inserted. At this point and beginning on one side of the transverse incision, a group of four sutures were very gently tensioned to close the gap without any of the sutures pulling out and then one suture only was tied. Without releasing the tension on the remaining three sutures, the fifth suture was gently tensioned and added to the remaining group, so that four tensioned sutures are still closing the gap. This process is repeated moving from one side of the extensor mechanism gap to the other side, sequentially tensioning and tying sutures to ensure an even distribution of tension with no suture cutting out, until the defect was completely closed. It must be emphasised that this suturing process was executed gently and slowly and this was critical to the success of this new procedure. A tourniquet had been applied high on the thigh and a completely bloodless field achieved by the use of an Esmark bandage.

Following patellectomy, the leg was immobilised in a removable extension splint (ProCare, DJ, LLC, Vista, CA 92081, USA). Patients were instructed by the physiotherapist to remove the splint twice a day to allow passive manually assisted range of motion exercises or active flexion strictly within the limits of pain (with a maximum range of 90°). Ambulation was in the splint, with the use of crutches if necessary, depending on the confidence of the patient for up to 6 weeks. Active closed kinetic chain exercises were introduced at this stage progressing on to open chain exercises after a further 2 weeks, starting with standing single leg lifts and leading on to unrestricted activity by 3 months. The initial rehabilitation was documented, and patients were reviewed at 2 weeks, 3 and 6 months post surgery.

Patients were assessed using the Lysholm knee [14] scoring system and visual analogue score for pain (VAS), ten being the worst pain, and zero, no pain at all. The current exercise level and active range of motion was recorded. Knee extension force was measured using Keiser 1131 Air Resistance Physical Trainer. One patient who had undergone patellectomy 16 years ago underwent an arthroscopy for swelling, knee pain and giving way. The arthroscopic findings are discussed.

## Results

Between 1996 and 2007, eight patellectomies were undertaken in six patients for idiopathic osteoarthritis. The patient demographics are shown in Table 1. All patients had undergone a previous arthroscopy that had confirmed the location of arthritis, either on the patella, trochlea or on both sides. There was one male and five female patients, with a mean age of 41.13 years (SD 8.5). Two patients underwent staged bilateral patellectomy one year apart. The mean follow-up was 11 years (SD 3.63).

**Table 1:** Demographics and outcomes

	Age at surgery/sex	VAS score before operation	Occupation	Follow-up (years)	Disease location	VAS score operated knee/s	VAS score contralateral knee	Lysholm score	Active ROM degrees (non-operated knee)	Force (*N*) (% of non-operated knee)
1	49/F	10	Nurse	16	Patella + trochlea	5	2	60	0–135 (0–135)	158 (87)
2	32/F	10	Labourer	12	Patella + trochlea	4	8	49	0–135 (0–135)	181 (115)
3	39/F	10, 10	Clerical Officer	12	Patella + trochlea	7, 8	–	29	0–110 (0–110)	249 (249)
4	47/F	10	Office work	11	Patella	0	0	85	0–135 (0–135)	350 (100)
5	31 and 32/F	10, 10	Environmental scientist	8	Patella	0, 0	–	56	10–95 (0–110)	181 (249)
6	51/F	10	Physiotherapist	5	Patella	0	0	91	0–135 (0–145)	408 (100)

At postoperative follow-up (range 5–16 years), the active range of movement, pain relief, outcome score, and visual analogue scale (VAS) at follow-up are given in Table 1. Patients with patella-only disease had excellent pain relief. They scored better on the VAS (0) and Lysholm score (average 77 SD 19) than those with patella and trochlear disease (VAS 6 SD 1.8 and Lysholm 44 SD 16). In addition, the maximum extension force was greater in patients with patella-only disease. Patients who had the longest follow-up tended to have worse outcomes; it is not clear whether this is age-related or demonstrates some deterioration over time.

**Fig. 2:**
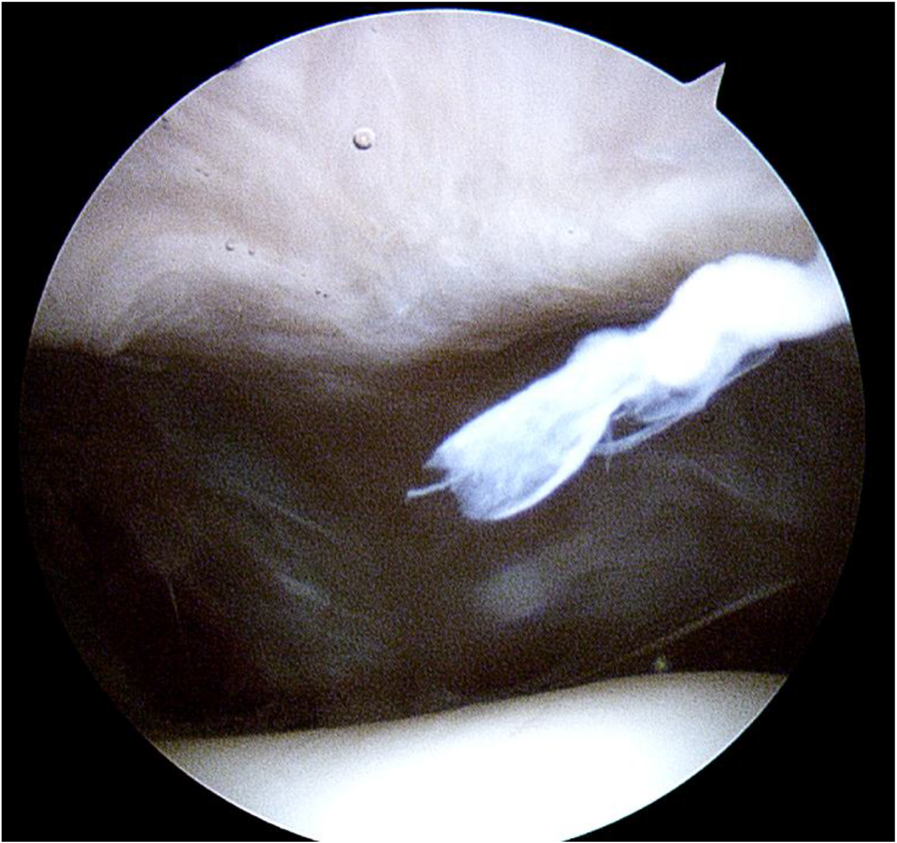
Arthroscopy 16 years following patellectomy. A well-healed patella tendon and quadriceps tendon anastomosis is seen

One patient (case 1) underwent arthroscopy 16 years following patellectomy (Fig. 2) for symptoms of giving way and swelling in the left knee (she had undergone bilateral patellectomies). An MRI scan showed oedema at the patella tendon/quadriceps tendon junction. An arthroscopy was performed which revealed a normal tendon junction but ICRS grade 2 arthritis in the trochlea, and this was debrided (Fig. 2). At follow-up, her symptoms had improved significantly. She had an excellent range of movement in the patellectomised knee of 0–135° compared with the contralateral knee 0–140°, and was able to extend her knees against 158 N of resistance symmetrically (87 % of the contralateral knee).

**Fig. 3:**
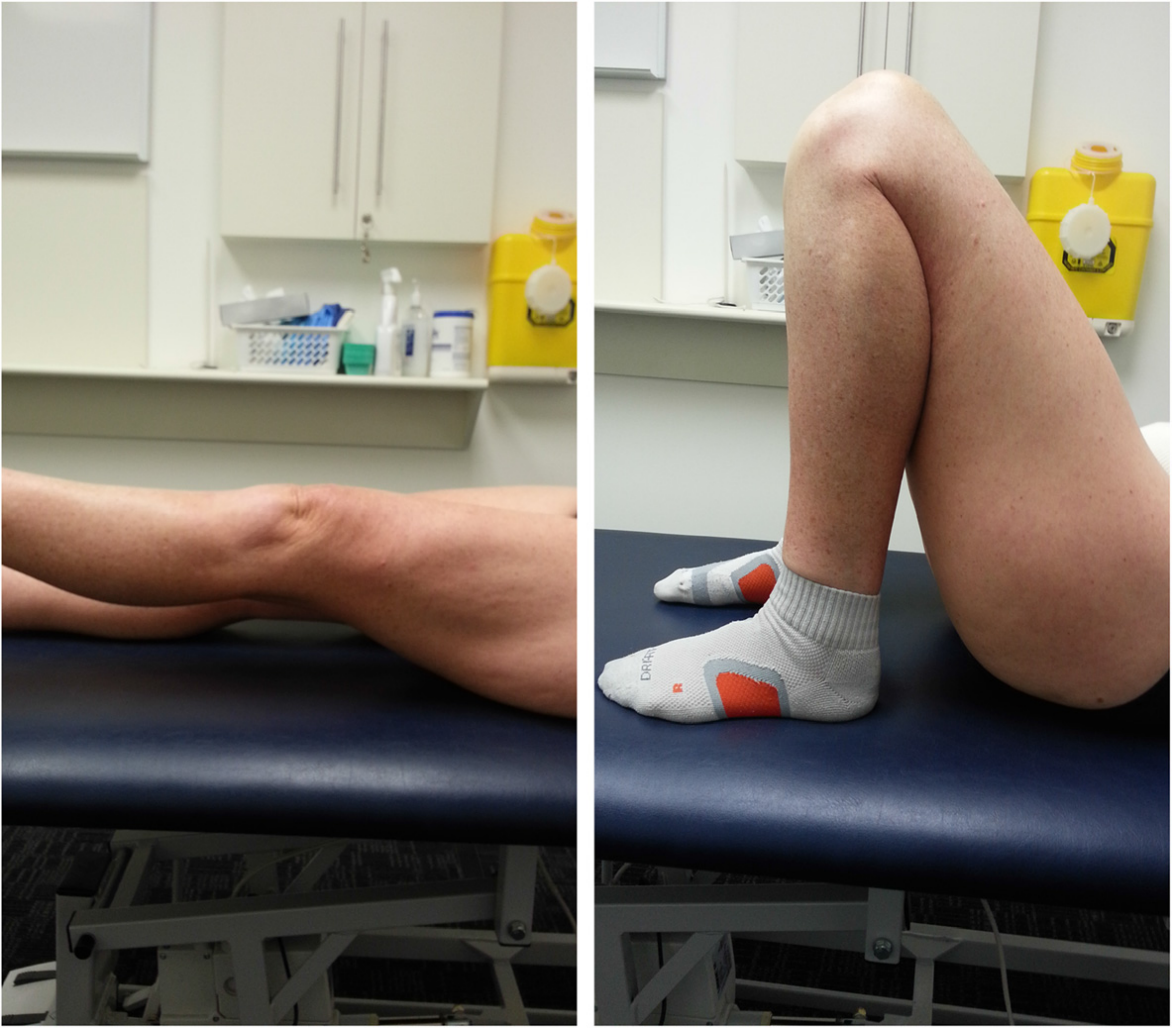
Clinical photograph of patient no. 6. Photograph illustrates active straight leg raise and full flexion following patellectomy of the left knee 5 years following surgery

Another patient (case 6) was tested for knee extension strength (Air300 Leg extension, Keiser, Fresno, CA 93706, USA); she obtained 100 % of the strength of her normal contralateral leg, with a range of motion of 0–135° (compared to 0–145° in the contralateral leg). Figure 3 shows a clinical photograph of case 6 with active straight leg raise and full knee flexion.

## Discussion

Patellectomy is only one of a number of surgical treatments available for the management of patellofemoral osteoarthritis. These include soft-tissue realignment procedures, osteotomies of the tibial tubercle, chondral regeneration procedures, patellofemoral replacement and total knee arthroplasty (TKA) [14].

The technique we have described differs from previous reported surgical techniques as it shortens the extensor mechanism by the vertical height of the patella and thus compensates for the reduced lever arm of the now absent patella by tensioning the extensor mechanism. In addition, the direct end-to-end anastomosis seems to create a larger tissue mass than other techniques, making the cosmetic appearance and condylar impact protection better. In 1969, Castaing reported a series of 17 patients who had undergone patellectomy through a vertical incision over the patella. The patella was removed and a full thickness quadriceps flap was turned down and sutured to the patellar tendon with the medial and lateral retinacula sutured over the turn down. Six of the patients reported good to excellent results [15]. Compere described a technique in 29 patients, where fibres on the dorsum of the patella were preserved, but the patella was everted and enucleated. The medial and lateral borders of the patella tendon and quadriceps were sutured together to fashion a tube. Patients had a mean age of 43.5 years. Although 90 % of patellectomies had an excellent result, he commented that quadriceps strength, and extensor lag were problematic [4].

Steurer et al. described a cruciform technique to approach the patella; however, results were no different to conventional techniques [16, 17]. Baker and Hughston reported on the outcomes of patellectomy using the Miyakawa technique where a partial thickness strip of quadriceps tendon is turned down over the void left by the patella and the vastus lateralis and medialis are advanced, crossed over each other, and attached to the quadriceps tendon. They reported 19/20 patients were satisfied [18].

Active extension was excellent with seven out of eight patellectomies having full active extension. Flexion was also good. Lysholm score results were fair when disease was confined to the patella. Patients who had disease of both patella and trochlea had poor scores [14]. Consistent with our findings, Professor Bentley and colleagues reported an 82 % satisfaction rate following patellectomy for severe chondromalacia (isolated patella arthritis) [19]. He also advised that outcomes following patellectomy for patellofemoral (patella and trochlea) osteoarthritis were less favourable [20]. Unfortunately, Lysholm scores were not available for patients prior to surgery.

One of our patients (case 6) who performed well was a physiotherapist. She took 2 years to get back to her normal activity level. Her exercise programme is detailed in Table 2. Case 3 had patellofemoral disease and was not compliant with the recommended rehabilitation protocol. She had ongoing pain and an inferior outcome.

**Table 2:** Recommended rehabilitation protocol

Period after surgery	Immobilisation	Exercise
0–2 weeks	Extension splint	Ice (6/52) post exercise as needed
		Anti-inflammatory medication (6/52)
		Gluteal and calf muscle exercises, while leg in splint
		NWB until 6/52, passive flexion as pain allows
		Do not load quads for 6 weeks—specific instruction!
		Maintain cardiovascular fitness
2–4 weeks	Extension splint	Start rowing machine—keep affected leg straight over the edge of the rower in splint
		Ice and passive ROM
		Intermittent passive flexion
		Swimming with drag float
		No active leg extension first 6 weeks
6 weeks	Remove extension splint	Allow full active extension
		Full flexion
		Aim to discontinue splint
6–12 weeks	Nil	Active/assisted quadriceps extensions
		Gradually increase loading over second 6 weeks
		Full active ROM swimming
12–20 weeks	Nil	5-km walking/running daily on hills as pain allows
>20 weeks	Nil	Commence leg presses
		Lunges
		Squats

Rehabilitation is an important feature relating to improved outcomes: the best outcomes were associated with intensive rehabilitation. The proposed rehabilitation protocol (Table 2) was derived from that used by the best performing patient (case 6). Patellectomy is an operation that requires extensive rehabilitation and can have good results in carefully selected patients.

**Fig. 4:**
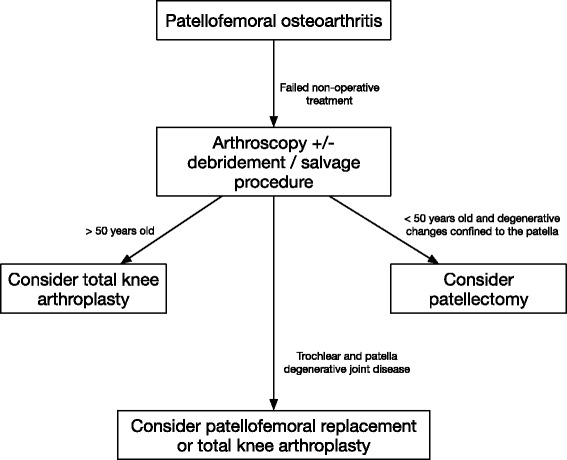
Algorithm for the surgical management of patellofemoral arthritis

The mean age of our patients was 41 years; however, there is no specific age limit for patellectomy. We would advise that TKA is less appealing in patients under the age of 50, whereas patellectomy is more appealing given that resurfacing arthroplasty does not work well. The procedure should not be regarded as a first line and should only be considered after debridement or other salvage procedures have failed. Degenerative disease of the trochlear is a relative contraindication for patellectomy. Undertaking a patellectomy procedure is not without risk and may have serious consequences if it fails; the patient must be counselled regarding this. An algorithm for the surgical management of patellofemoral arthritis is present in Fig. 4.

Patients may develop progressive osteoarthritis and require a TKA following patellectomy; however, this has not been required in our series. TKA following patellectomy via a direct midline approach is perfectly practical, and no attempt to reconstruct the patella would be anticipated to be needed.

The limitations in this case series are the small sample size and the lack of preoperative functional scores. Severe isolated patella osteoarthritis in young patients is relatively rare, and the number of patients willing to consider patellectomy is low; hence, we only recruited eight cases in six patients over 11 years despite the busy metropolitan practice of a dedicated specialist knee surgeon (GK).

Strengths of this study include the long-term follow-up with no loss to follow-up and the standardised surgical technique associated with a single surgeon series. A larger prospective series with comparative data between isolated patella versus patellar and trochlear involvement with preoperative and postoperative patellofemoral outcome scores would be of interest in further evaluating this new technique.

Although patellectomy has been termed a ‘salvage procedure’, we believe that it avoids the morbidity associated with other techniques and should be considered in young patients with severe patellar-only osteoarthritis.

## Conclusion

Patellectomy is an historical surgical treatment for osteoarthritis of the patellofemoral joint that has become unpopular in recent times because of poor outcomes. We suggest that patellectomy, using the described tension-preserving technique, can produce excellent results in young patients who have disease confined to the patella and are motivated to undergo appropriate rehabilitation.

## Abbreviations

VAS: visual analogue scale
